# Gender-Dependent Cognitive and Metabolic Benefits Due to Glyoxalase 1 (Glo1) Overexpression in Age-Accelerated SAMP8 Mice

**DOI:** 10.3390/antiox14080946

**Published:** 2025-08-01

**Authors:** Alcir Luiz Dafre, Taketo Taguchi, Yelena Dayn, Antonio Currais, Pamela Maher

**Affiliations:** 1 Cellular Neurobiology Laboratory, Salk Institute for Biological Studies, La Jolla, CA 92037, USA; 2 Biochemistry Department, Federal University of Santa Catarina, Florianópolis 88040-900, SC, Brazil; 3 Transgenic Core Facility, Salk Institute for Biological Studies, La Jolla, CA 92037, USA

**Keywords:** glyoxalase, methylglyoxal, SAMP8, geroscience, survival, oxytosis/ferroptosis, cognitive function

## Abstract

As the size of the elderly population increases, the need for an improved understanding of what leads to the age-related decline in physiological function continues to grow. SAMP8 mice were selected for their accelerated aging phenotype. The low levels of glyoxalase 1 (Glo1), the main enzyme that removes the reactive dicarbonyl methylglyoxal (MGO), in the cerebral cortex of SAMP8 mice prompted us to produce the first transgenic mice overexpressing Glo1 against the SAMP8 background, aimed at rescuing the accelerated aging phenotype. Selected health and biochemical endpoints were assessed in ten-month-old SAMP8 mice overexpressing Glo1. Glo1 overexpression increased median survival in males (21%) and females (4.6%), which was associated with better memory performance. Glo1 overexpression also increased synaptic markers (synaptophysin and SNAP25) as well as markers of mitochondrial function (NDUFB8, SDHB) and negative modulators of oxytosis/ferroptosis (NQO1, FTH1, and GPx4) in the cerebral cortex. For all parameters analyzed, the effect of Glo1 overexpression was more pronounced in males. Overall, the data support the beneficial effects of overexpressing Glo1 in multiple tissues, especially in SAMP8 males, suggesting a possible gender effect of MGO in aging. Both modulation of oxytosis/ferroptosis and mitochondrial metabolism warrant further investigation as potential mechanisms underlying the improved health span of Glo1 mice.

## 1. Introduction

Methylglyoxal (MGO) plays a significant role in numerous age-related diseases, including neurodegeneration [1]. This highly reactive dicarbonyl is an endogenously produced byproduct of glycolysis that interacts with basic amino acids of proteins leading to the formation of adducts [1]. One of the main resulting products, MGO hydroimidazolone, binds with high affinity to RAGE [2], the receptor of advanced glycation end-products (AGEs), activating pro-inflammatory and oxidative pathways, which have been associated with mitochondrial dysfunction and neuronal damage [3].

Glyoxalase 1 (Glo1) is the rate-limiting enzyme in MGO detoxification [4]. After reacting non-enzymatically with glutathione (GSH), the MGO-GSH conjugate becomes a substrate for Glo1, generating S-D-lactoylglutathione (SDL), which is further metabolized by glyoxalase 2 (Glo2), generating D-lactate, while GSH is regenerated in the process [5,6]. Glo1 inhibitors are actively being tested for cancer therapy, as many cancer types present elevated rates of glycolysis [7], a condition known to augment MGO formation and toxicity, where Glo1 activity improves cancer cell survival [6,8]. Conversely, overexpression of Glo1 has shown beneficial effects on the cardiovascular system, such as reducing infarct size in ischemic stroke in mice [9], improving cognitive indexes and neurovascular coupling in a mouse model of type 1 diabetes [10], and attenuating hyperglycemia-induced endothelial dysfunction and oxidative damage in the diabetic rat heart [11].

Recent evidence highlights the importance of Glo2 in the lactylation of proteins, a post-translational modification of basic protein residues by lactate [12,13]. Augmented MGO production, such as in diabetes, would favor SDL buildup, and thus D-lactylation of proteins (DKlac) [12,14]. Immune response, histone tail modification, and modulation of the tumor microenvironment were recently highlighted as relevant processes modulated by lactylation [13,14,15]. Thus, we reasoned that Glo1 overexpression might also increase SDL, the Glo2 substrate, promoting lactylation of proteins.

The role of Glo1 in anti-aging has been demonstrated with *C. elegans*, where ablation of bacteria-derived MGO or overexpression of glyoxalase increased the *C. elegans* lifespan [16]. Exogenous administration of MGO leads to a decline in memory and increased depression-like behavior [17]. In addition, decreases in Glo1 activity were associated with neuroinflammation and oxidative stress [18] along with the activation of RAGE [18,19] and elevated AGE levels [20]. Moreover, since MGO interferes with brain insulin signaling and glucose utilization, thereby exacerbating metabolic stress, MGO can potentially promote the onset of age-related cognitive decline [21,22]. Furthermore, in humans, higher plasma MGO levels correlated with poorer memory performance and reduced grey matter volume, further supporting a role for MGO in age-related cognitive decline [23,24].

Given that Glo1 shows reduced activity in neurodegenerative diseases, leading to increased accumulation of MGO and AGEs in brain tissue [25], which has been correlated with cognitive decline in humans and rodents [21,22,23,24], and that previous data from our laboratory identified low levels of Glo1 in the brains of age-accelerated SAMP8 mice [26], we asked if Glo1 overexpression (Glo1 OEX) would modify the aging phenotype of this mouse model.

Oxytosis/ferroptosis is a form of regulated cell death characterized by the iron-dependent accumulation of lipid peroxides [27,28]. This pathway is closely related to the pathophysiological processes of many age-linked diseases, including nervous system and blood diseases [29,30]. Oxytotic/ferroptotic events that occur when cells in culture are stimulated with pro-oxytotic/ferroptotic agents such as RSL3 and erastin [30,31] can be observed during aging, however, at sublethal levels and in a time frame of years to decades [27]. A number of recent publications indicate that the accelerated aging phenotype in SAMP8 mice is associated with oxytosis/ferroptosis where several anti-aging compounds counteract the neurodegeneration process by inhibiting the development of oxytosis/ferroptosis markers [30,32,33,34,35,36]. Most of these studies highlight the importance of Nrf2 in controlling the expression of several anti-oxytotic/anti-ferroptotic genes/proteins, such as glutathione peroxidase 4 (GPx4) and ferritin heavy chain 1 (FTH1). Therefore, we also investigated whether one way that Glo1 OEX could be beneficial in the context of aging was by modulating the expression of these proteins.

## 2. Materials and Methods

### 2.1. Animals

The objective of this study was to evaluate the potential benefits of overexpressing Glo1 in the aging-accelerated SAMP8 mice.

All studies were carried out in accordance with the recommendations in the Guide for the Care and Use of Laboratory Animals of the National Institute of Health. The protocol was approved by the Institutional Animal Care and Use Committee of the Salk Institute for Biological Studies (12-00001; approval 10 March 2021). Mice were housed in an environmentally controlled vivarium with lights on from 06:00 to 18:00 h, temperature at 20–23 °C, lighting at 300 lux (less than 50 lux in the cages), and humidity at 40–60%. Cage cleaning was not performed on the same day of behavioral testing. Food and water were available ad libitum.

### 2.2. Generation of Mice Overexpressing Glo1 Against the SAMP8 Background

#### 2.2.1. SAMP8 ES Cell Lines Established

The SAMP8 ES cell lines were de novo derived from the inner cell mass (ICM) of the SAMP8 blastocyst-stage mouse embryos.

#### 2.2.2. Rosa26-CAG-cMyc-hGlo1-IRES2-tdTomato Targeting Vector Construction

The targeting construct was generated using a molecular cloning approach. The Ai65 (RCFL-tdT) targeting vector (plasmid#61577) was obtained from Addgene (Watertown, MA, USA). The Ai65 (RCFL-tdT) targeting vector was digested with restriction enzymes BstBI and MluI to remove the RCFL-tdT cassette. Using NEBuilder^®^HiFi DNA Assembly Master Mix (NEB, E2621S), cMyc-hGlo1 coding cDNA, IRES2, and tdTomato coding cDNA were assembled into the digested Ai65 vector to generate the final targeting vector: Rosa26-CAG-cMyc-hGlo1-IRES2-tdTomato (Figure S1a).

#### 2.2.3. Generation of Rosa26-CAG-cMyc-hGlo1-IRES2-tdTomato Mice

The Rosa26-CAG-cMyc-hGlo1-IRES2-tdTomato targeting vector contained 5′and 3′ Rosa26 homology arms, as well as a PGK-Neo cassette for positive selection. The targeting vector was linearized and electroporated into the SAMP8 ES cell line using a BioRad electroporator (Hercules, CA, USA). G418-resistant ES clones were screened by Southern blotting, PCR, Western blotting, and sequencing combined analysis. For Southern blot analysis, genomic DNAs from targeted ES clones were digested with EcoRV restriction enzyme and then probed with a probe for the 5′ arm (Figure S1b). PCR primers were designed according to the expected insertion pattern. The 5′ arm forward primer was designed according to the wild-type sequences of the 5′ arm (5′-cttccctcgtgatctgcaactc-3′), and the reverse primers were designed according to the insertion sequences of either the CAG promoter (5′-gatgtactgccaagtgggcag-3′) for the targeted allele or the 3′ arm (5′-ccagatgactacctatcctcccat-3′) for the wild-type allele (Figure S1c). Positively targeted ES cells were injected into C57BL/6 mouse blastocysts to generate chimeric mice. Chimeric mice then were bred with SAMP8 mice to test for germline transmission and generate F1 heterozygous mice. These mice were then used to generate Glo1 OEX homozygous mice, which were used in all of the analyses shown.

### 2.3. Survival Curves

Wild-type (WT) and Glo1 OEX SAMP8 mice (male: N = 35–37; female: N = 25–27) were used for lifespan analysis. All mice were kept under standard housing conditions without any additional intervention. Mice that became severely ill or were recommended for euthanasia by the veterinarian were euthanized and marked censored in the survival curves.

### 2.4. Novel Object Recognition

Ten-month-old mice were used for these studies. Each mouse was placed in an open arena for habituation for 10 min. In the training phase, two identical objects were placed in opposite corners of the apparatus. The mouse was placed in a corner without objects and locomotion recorded for 10 min. One hour after training, the test was performed by replacing one object with a novel object. The mouse was placed into the apparatus in the opposite corner to the objects and recorded for 5 min. The object recognition index was calculated as the fraction of the total exploratory time that the animal explored the novel object.

### 2.5. Acoustic Startle Response and Visual Cliff Test

Hearing loss was assessed in 10-month-old mice based on the acoustic startle response where the subject’s failure to respond to a sudden sound (a clicker) was accessed [37]. Vision loss was evaluated by the visual cliff test by assessing the inability to reach toward the ground when suspended by the tail [38]. Each deficit was rated using a simple scale: a score of zero indicated no deficit, 0.5 represented a mild deficit, and 1.0 indicated a severe deficit.

### 2.6. Western Blot

Tissue samples were mechanically homogenized with a TissueRuptor (Qiagen, Hilden, Germany) and the nucleus, mitochondria, and cytoplasm were separated by the Reap+ method [39]. Briefly, after homogenizing in PBS, samples were centrifuged for 10 s 15,000× *g*. The pellet was used for nuclear preparation, the supernatant for cytosolic, and mitochondrial fractions after a 10,000× *g* centrifugation (4 °C, 15 min). Protein was assayed by the BCA method (Invitrogen, Waltham, MA, USA).

Ten-month-old WT and Glo1 OEX male mice were probed for Glo1 to confirm the success of the construct, and for Glo2 to verify if Glo1 overexpression had any effect on Glo2 protein levels. The Glo1 expression was also compared between WT SAMP8 males and females. To investigate if WT SAMP8 mice present lower Glo1 levels in multiple tissues, not only in the brain as previously reported [26], C57/BL6 mice of the same age were used as the WT strain control.

Samples were diluted in sample buffer (66 mM Tris/HCl pH 6.8, 10% glycerol, 2% SDS, 5% 2-marcaptoethanol, 0.1% bromophenol blue) and analyzed by SDS-PAGE using 4–12% gradient gels (Criterion XT Precast Bis-Tris Gels, Bio-Rad, Hercules, CA, USA). Stain-free gels were used to quantify proteins and as a loading control. Proteins were transferred to polyvinylidene fluoride membranes and tested with the indicated primary antibodies. Horseradish peroxidase-conjugated secondary antibodies were diluted 1/5000 in 2% skim milk in tris-buffered saline/0.1% Tween 20 prior to use. Images were obtained by the Chemidoc MP Imaging System (Bio-Rad) and quantified with the ImageJ software, version 1.54d, available at https://imagej.net/ij/ (accessed on 26 July 2025).

The following antibodies were used at the indicated dilutions: GPx4 (1:3000; #52455), synaptosome-associated protein of 25 kD (SNAP25; 1:3000; #4117), synaptophysin (SYP; 1:1000; #42406), voltage-dependent anion channel (VDAC; 1:10,000; #4866), and FTH1 (1:20,000; #4393) were from Cell Signaling (Danvers, MA, USA); mitochondrial electron transport chain (ETC) markers (1:10,000; #110413) including members of the complexes CI (NDUFB8, NADH:ubiquinone oxidoreductase subunit B8), CII (SDHB, succinate dehydrogenase B), CIII (UQCRC2, ubiquinol-cytochrome c reductase core protein 2) and CV (ATP5A, ATP synthase mitochondrial F1 complex subunit alpha) and histone 3 (H3; 1:3000; #ab24834) were from Abcam (Waltham, MA, USA); postsynaptic density protein-95 (PSD95; 1:100; #MAS-046) was from Invitrogen (Carlsbad, CA, USA); DKlac (1:3000; PTM-1429) was from PTM Bio (Chicago, IL, USA); and Glo1 (1:3000 or 1:10,000; #SC6735), Glo2 (1:3000; #SC51092), and NAD(P)H quinone oxidoreductase 1 (NQO1; 1:500; #27111) were from Santa Cruz Biotechnology (Dallas, TX, USA).

### 2.7. Glyoxalase 1 Activity

Tissues from ten-month-old WT SAMP8 and Glo1 OEX mice tissues were homogenized as described for Western blotting, except that sample buffer was not added, and they were kept in PBS before being assayed for Glo1 activity. For the assay of Glo1 activity, the linear appearance of the product S-D-lactoylglutathione (2860 M^−1^ cm^−1^) was followed for 3 min, at 240 nm, in a 96-well plate reader [40]. The substrate, hemithioacetal, was formed nonenzymatically from the reaction of MGO (2 mM) and GSH (2 mM) in potassium phosphate buffer (50 mM, pH 6.6) for 10 min at 37 °C and immediately used in the assay.

### 2.8. Statistical Analysis

Unless otherwise stated, the studied parameters were assessed independently in male and female mice at 10 months of age. Endpoints were pairwise compared within males or females by the Student *t*-test, and gender effects were inferred by specific responses to Glo1 overexpression. Survival curves were computed based on a sample size of 37/35 (WT/Glo1) males and 25/27 females. Kaplan–Meier curves were plotted in GraphPad 8.0 (Boston, MA, USA) and pairwise comparisons made by the log-rank test. Unless stated, the sample size for Western blots was 6 animals. For other endpoints, the sample size is indicated in the figure legends.

## 3. Results

### 3.1. Characterizing the SAMP8 Mice Overexpressing Glo1

**Figure 1:**
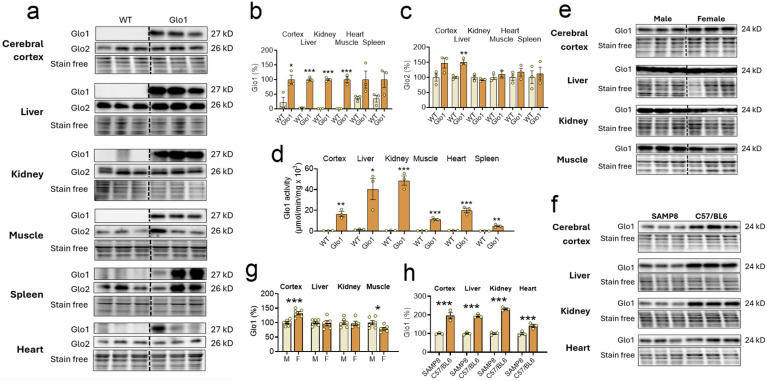
Background levels and overexpression of Glo1 in the aging-accelerated SAMP8 mice. (**a**) Tissues of ten-month-old wild-type (WT) and Glo1 OEX male SAMP8 mice were probed for Glo1 and Glo2 content with the corresponding antibodies, and respective quantification for Glo1 (**b**) and Glo2 (**c**) (N = 3). (**d**) Tissues of ten-month-old WT and Glo1 OEX male mice were assayed for Glo1 activity (N = 3). (**e**) Gender differences in Glo1 expression in tissues of 10-month-old WT SAMP8 mice (N = 6) and its respective quantification (**g**). (**f**) In order to compare the WT SAMP8 strain basal expression of Glo1 protein, the tissue samples of WT mice, as described in (**a**), were compared with samples obtained from WT C57/BL6 mice of the same age (N = 3). Their respective quantification is presented in (**h**). In the Western blots Glo1 presents a band at 28 KD and Glo2 at 26KD. Data are presented as individual values (circles) and mean ± SEM. Statistically significant differences are illustrated at * *p* < 0.05, ** *p* < 0.01, and *** *p* < 0.001.

To test the possible implications of low levels of Glo1 for the aging phenotype of SAMP8 mice, we generated for the first time mice ubiquitously overexpressing Glo1 across all tissues under the control of the c-Myc promoter, which was confirmed by Western blotting (Figure 1a), and performed respective quantification (Figure 1b). All tissues tested presented elevated levels of Glo1 in the Glo1 OEX mice, as compared to the WT SAMP8 mice, including the cerebral cortex, liver, kidney, skeletal muscle, and spleen. Due to the very strong signal resulting from the high expression of Glo1 protein in the Glo1 OEX mice, the detection of Glo1 in the WT mice was precluded when analyzed on the same blot. Therefore, we considered values obtained with Glo1 OEX mice as 100%. Band intensities higher than 10% in the WT animals, as shown in Figure 1b, are likely nonspecific, as no clear bands can be seen in the blots (Figure 1a), especially in the case of the heart and spleen. Importantly, the same elevated levels of Glo1 in the Glo1 OEX SAMP8 mice were demonstrated by assaying Glo1 activity (Figure 1d). The results showed 18–70-fold higher Glo1 activity in the Glo1 OEX mice as compared to the WT SAMP8 mice, as detected in the cerebral cortex, liver, kidney, muscle, heart, and spleen. The same pattern of increased expression of Glo1 in the Glo1 OEX SAMP8 mice was confirmed by immunohistochemistry in several tissues, including the brain, liver, kidney, heart, lung, and skeletal muscle (Figure S2a–f). Overexpression of Glo1 appears not to affect Glo2 levels in the same tissues (Figure 1a,c), except for the liver, where about 50% higher levels were observed.

We also noted gender differences in the levels of Glo1 expression in the SAMP8 mice (Figure 1e,g). While Glo1 expression was higher in the female (+33%) as compared to the male cortex, lower levels of Glo1 were found in female skeletal muscle (−20%). The liver and kidney presented no gender differences.

In a previous study, we reported that 10-month-old aging-accelerated male SAMP8 mice presented low levels of Glo1 in the cerebral cortex as compared to control SAMR1 mice of the same age [26]. We first confirmed this finding with a different strain of age-matched control mice and showed that multiple organs in C57/BL6 male mice showed at least two times higher Glo1 levels in the cerebral cortex, liver, and kidney, while in the heart, the levels were 41% higher (Figure 1f,h), as compared to 10-month-old SAMP8 male mice. Combined with the results from Figure 1e,f, it can be deduced that 10-month-old SAMP8 mice of both sexes present low background levels of Glo1 in multiple tissues, given that the gender differences were in the range of 20–30% but about a 2-fold difference was found between strains (SAMP8 vs. C57/BL6) in multiple tissues.

Multiple parameters were evaluated in the 10-month-old SAMP8 Glo1 OEX as an initial health assessment. Glo1 OEX mice blood biochemistry (Figure S3) presented no alteration, as compared to the WT animals, and was considered within the expected range. The locomotor activity (Figure S4) and limb strength (Figure S5) were similar between WT and Glo1 OEX mice. A histopathological evaluation was performed on several tissues of 10-month-old WT and Glo1 OEX SAMP8 mice (Table S1). The brains of all the mice showed no pathological changes, while the other tissues examined showed minimal to mild alterations that did not differ significantly between WT and Glo1 OEX mice. Differences were observed between male and female mice, but these differences were generally not affected by Glo1 OEX. Importantly, the results indicate that Glo1 overexpression did not cause increased age-related damage to the tissues that were examined.

**Figure 2:**
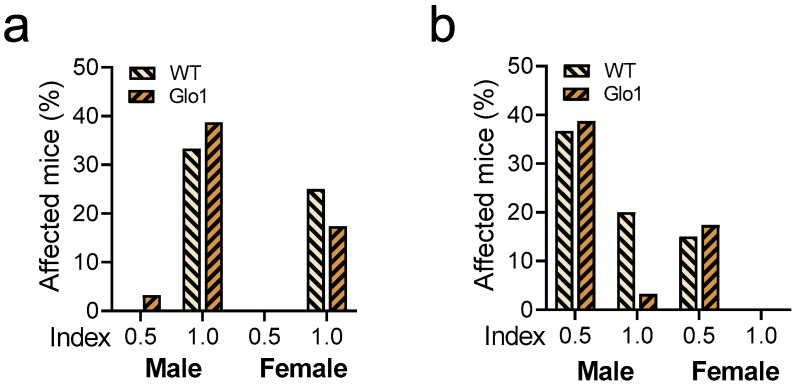
Hearing and vision loss in WT and Glo1 OEX SAMP8 mice. Acoustic startle (**a**) and visual cliff response (**b**) are presented for males and females. Responses were classified as normal (0.0), mild (0.5), or severe (1.0) impairment. The frequency distribution of categories 0.5 and 1.0 is presented in graphs. The number of animals was 30–31 for males and 20–24 for females.

Hearing loss and visual acuity were also evaluated in 10-month-old WT and Glo1 OEX SAMP8 mice. Hearing loss (Figure 2a) was observed in 33.3% (WT) and 41.9% (Glo1 OEX) of male mice, while the frequencies in females were 25% (WT) and 17.4% (Glo1 OEX). Thus, only marginal effects on hearing could be related to Glo1 overexpression. While Glo1 overexpression had a negligible effect on males presenting mild visual impairment (36.7% for WT and 38.7% for Glo1 OEX), the Glo1 OEX male mice showed a clear decrease in the fraction of mice with severe visual impairment (20% of WT males and 3.2% of Glo1 OEX males) (Figure 2b). Females were more resistant to age-related visual impairment (Figure 2b), as severe impairment was not observed in females, and only a low percentage of females scored in the mild impairment category, 15% (WT) and 17% (Glo1 OEX). Males presented about 3-fold more occurrences (56% WT and 42% Glo1 OEX), if compared to females that presented visual impairment.

**Figure 3:**
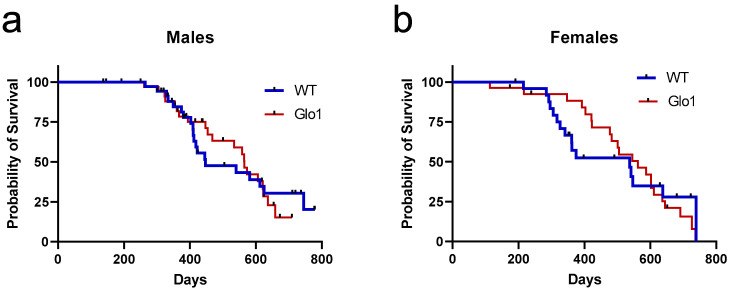
Survival curves of wild-type (WT) and Glo1 OEX male (**a**) and female (**b**) SAMP8 mice. Survival curves were computed based on a sample size of 37/35 (WT/Glo1) males and 25/27 (WT/Glo1) females.

The survival of the WT and Glo1 OEX SAMP8 mice was also examined (Figure 3). The log-rank test presented no differences in the long-term survival rates of male (Figure 3a) and female (Figure 3b) mice overexpressing Glo1. The male median survival was 447 days for WT SAMP8 mice and 565 days when Glo1 was overexpressed. The median survival for females was 537 days for WT and 562 for Glo1 OEX mice. Males showed an increase in median survival of 21.1% (Figure 3a) while females had a much lower increase (4.7%, Figure 3b). 

### 3.2. Memory and Synaptic Markers

**Figure 4:**
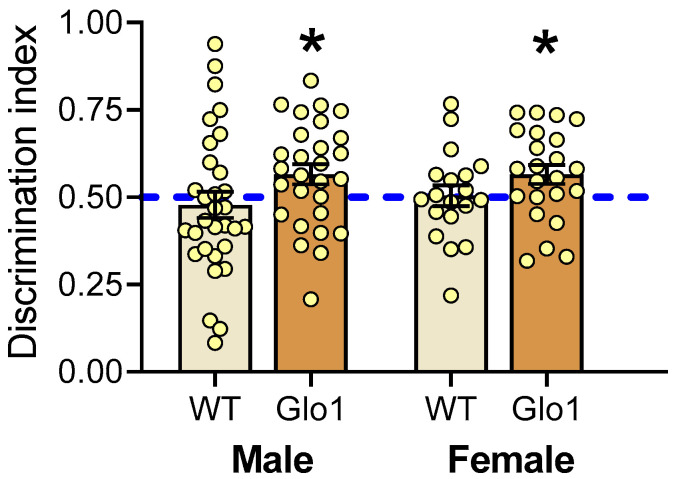
Novel object recognition test performed in male and female WT and Glo1 OEX SAMP8 mice. The discrimination index indicates the fraction of time mice spend with the novel object. Mice exhibiting a statistically significant preference for the novel object (exploration fraction > 0.5) were considered to have preserved spatial memory. Data are presented as individual values (circles) and mean ± SEM. Sample size was 19–23 for females and 28–31 for males. Statistically significant differences are illustrated at * *p* < 0.05.

To examine the effect of Glo1 OEX on cognitive function in the context of aging, the novel object recognition test (NOR) was performed at 10 months on both male and female mice (Figure 4). WT mice were unable to recognize the novel object, as they interacted with the novel object no more than 50% of the time, which is comparable to a random chance of interacting with the objects. Mice overexpressing Glo1 presented a discrimination index significantly higher than 50% for both sexes, suggesting memory was preserved in the Glo1 OEX mice.

**Figure 5:**
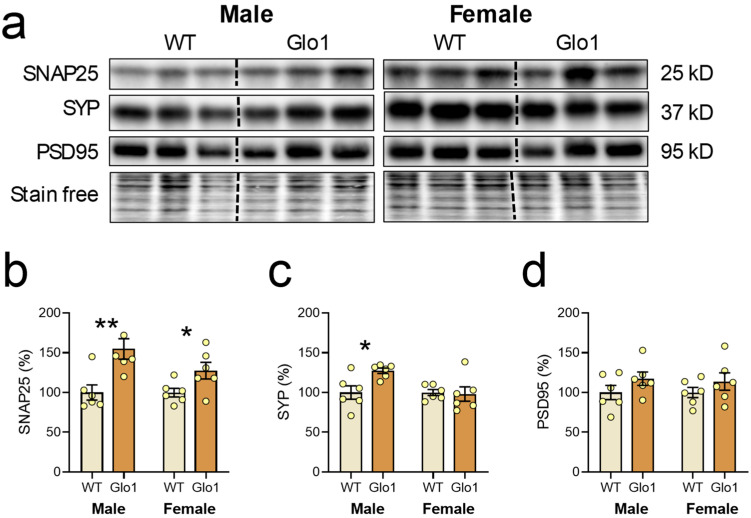
Synaptic markers in the cerebral cortexes of WT and Glo1 OEX male and female SAMP8 mice. (**a**) Representative blots of SNAP25, SYP, and PSD95 and their respective quantification (**b**–**d**). Data are presented as individual values (circles) and mean ± SEM (N = 6). Statistically significant differences are illustrated at * *p* < 0.05 and ** *p* < 0.01.

The number of synapses in the prefrontal cortex is known to decline during aging [41], and the abundance of synaptic markers is widely used as indirect evidence for synaptic density. For instance, environmental enrichment increases the levels of PSD95 and SYP, which was associated with a higher number of synapses and directly correlated with cognitive function [42,43]. To investigate a possible correlation between spatial memory performance of Glo1 OEX and the abundance of synapses, three synaptic markers were evaluated in the cerebral cortex using Western blotting of tissue extracts (Figure 5a). SNAP-25 is a presynaptic membrane protein that participates in the vesicular release of neurotransmitters [44]. SYP is a marker of synapse distribution participating in both endo- and exocytosis [45]. PSD-95 is a major regulator of synaptic maturation by interacting, stabilizing, and trafficking glutamate receptors to the postsynaptic membrane [46]. Quantification of these markers revealed that 10-month-old Glo1 OEX SAMP8 males showed increased levels of SNAP25 and SYP (Figure 5b,c), with PSD95 presenting only a tendency to increase (Figure 5d), as compared with WT males. The effect of Glo1 OEX on synaptic markers was weaker in females, with only a 27% increase in SNAP25 levels being observed.

### 3.3. Mitochondria Markers and Antioxidant Protection

**Figure 6:**
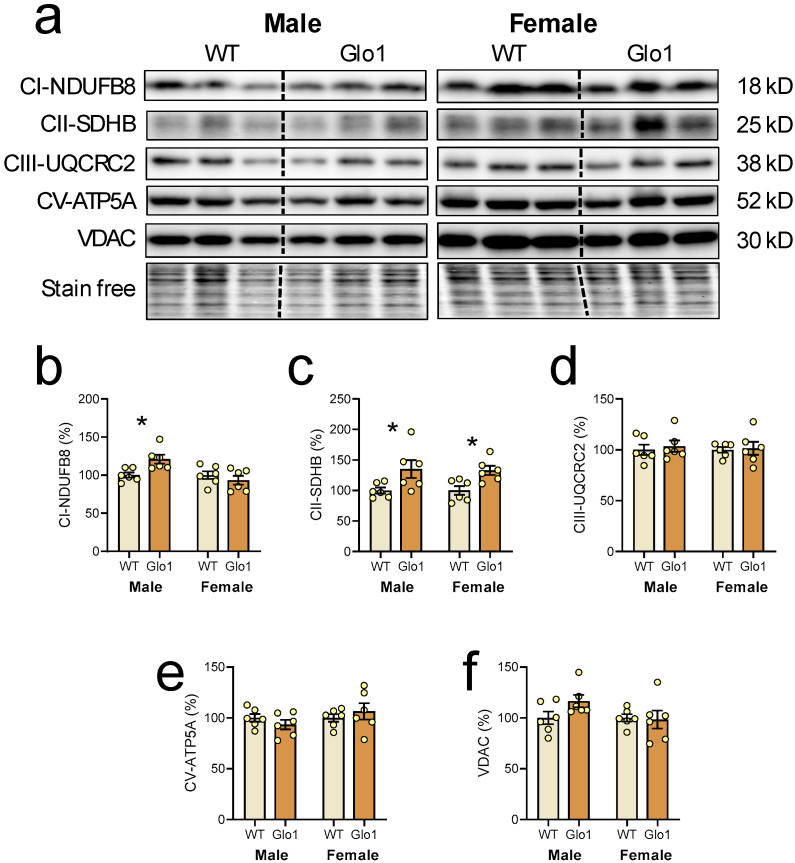
Mitochondrial markers in the cerebral cortex of 10-month-old WT and Glo1 OEX SAMP8 mice. Representative blot images (**a**) and their respective quantification for NDUFB8 (**b**), SDHB (**c**), UQRC2 (**d**), ATP5A (**e**), and VDAC (**f**). Data are presented as individual values (circles) and mean ± SEM (N = 6). Statistically significant differences are illustrated at * *p* < 0.05.

Since mitochondrial function is key to brain activity, mitochondrial markers were evaluated in the cerebral cortex of 10-month-old WT and Glo1 OEX SAMP8 mice (Figure 6a). The levels of NDUFFB8, a marker of complex I, were increased only in male Glo1 OEX mice relative to WT mice (Figure 6b), and a marker of complex II, SDHB, was increased in both males and females (Figure 6c). No differences were observed in the expression of complex III (UQCR2, Figure 6d), complex V (ATP5A, Figure 6e), and VDAC (Figure 6f) between the WT and Glo1 OEX mice.

**Figure 7:**
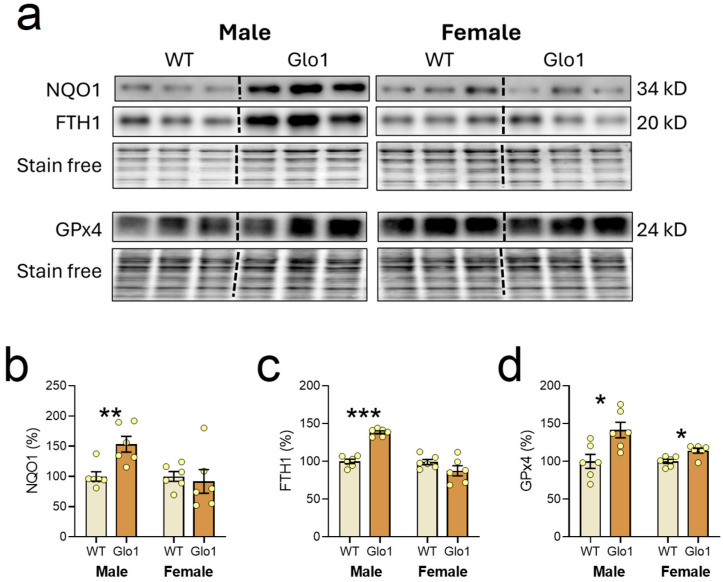
Transcription factor Nrf2 target genes expressed in the cerebral cortex of 10-month-old WT and Glo1 OEX SAMP8 mice. Representative blot images (**a**) and their respective quantification for NQO1 (**b**), FTH1 (**c**), and GPx4 (**d**). Data are presented as individual values (circles) and mean ± SEM (N = 6). Statistically significant differences are illustrated at * *p* < 0.05, ** *p* < 0.01, and *** *p* < 0.001.

The expression of Nrf2 transcription factor-related proteins was also evaluated in the cerebral cortex of 10-month-old WT and Glo1 OEX SAMP8 mice (Figure 7a). The quantification revealed an increase in the expression of NQO1 (Figure 7b), FTH1 (Figure 7c), and GPx4 (Figure 7d) in 10-month-old Glo1 OEX male mice while, for female Glo1 OEX mice, an increase was only observed for GPx4. Despite the clear increases in the expression of Nrf2 targets, Nrf2 levels in the nuclear fraction appeared unaltered between WT and Glo1 OEX SAMP8 mice of both sexes although there was a very high degree of variability preventing us from drawing a firm conclusion (males: 100.0 ± 18.9 WT and 90.5 ± 71.9% Glo1 OEX; females: 100.0 ± 62.6% WT and 99.1 ± 47.7% Glo1 OEX. N = 6). Nrf2 presents multiple control mechanisms, including degradation, phosphorylation, import to the nucleus, and regulation by interacting proteins, among others [47,48], so we cannot rule out the participation of Nrf2 in regulating the expression of NQO1, FTH1, and GPx4. Alternatively, other transcription factors could be associated with the observed increases in NQO1, FTH1, and GPx4.

**Figure 8:**
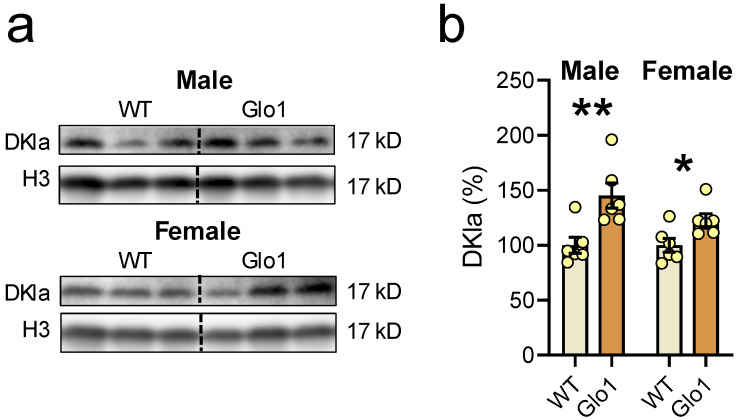
Glo1 OEX increased the D-Lactylation of lysine residues (DKla) in the nuclear extract. (**a**) Blot images of DKla and histone 3 (H3) as a loading control in males (upper panel) and females (lower panel), and their respective quantification (**b**). Data are presented as individual values (circles) and mean ± SEM (N = 6). Statistically significant differences are illustrated at * *p* < 0.05 and ** *p* < 0.01.

In theory, Glo1 OEX would result in more efficient metabolization of MGO, resulting in increased SDL, which would be able to produce D-lactylation of histones. When the nuclear extract was tested, the antibody recognizing the “D” isomer of lactoyl lysine (DKla) produced a clear band at 17 kD (Figure 8a), corresponding to the migration position of H3, which was used as the loading control. The band intensity was higher in male as well as in female Glo1 OEX mice (Figure 8b), when compared to the WT SAMP8 mice, likely representing H3 D-lactylation.

## 4. Discussion

Lower Glo1 levels have previously been reported in the cerebral cortex [26] and liver [49] of SAMP8 mice, which was associated with increased levels of AGEs and RAGE [49,50]. This information, albeit limited, suggests that MGO is a good candidate for contributing to the accelerated aging phenotype of SAMP8 mice, which is characterized by cognitive decline, recapitulating mechanisms found in sporadic Alzheimer’s disease [51]. Thus, we hypothesized that Glo1 overexpression would bring benefits by limiting the MGO burden and consequently restraining the aging process of SAMP8 mice, particularly in their brains. Nevertheless, a limitation of our work is that we did not look at the levels of free MGO or MGO-protein adducts. Thus, we cannot determine a direct functional relationship between the beneficial effects of Glo1 overexpression on aging and MGO levels. Additionally, the downregulation of Glo1 in the SAMP8 mouse background could provide further evidence for the anti-aging role of Glo1 but is beyond the scope of these studies.

Glo1 overexpression in the SAMP8 background did not show any noticeable negative effects on blood-based clinical indexes, locomotor activity, muscle strength, or routine clinical histopathology assessments. Moreover, there were multiple indications that Glo1 overexpression provided benefits to 10-month-old SAMP mice, including a lower frequency of severe visual impairment, increased median survival in males, and better performance in the NOR for both males and females. As compared to SAMR1 [26] and C5/BL6 mice (this work), the SAMP8 strain presents intrinsically low levels of Glo1 in cerebral cortex; therefore, the presented benefits of Glo1 OEX may be related to mitigating the effects of MGO. Furthermore, as compared to females, WT SAMP8 males present lower levels of Glo1 in their cerebral cortex; thus, overexpression of Glo1 might be one reason for the greater benefits seen in males. We speculate that this lower level of Glo1 in multiple organs in SAMP8 mice, and especially in the male brains, has a significant impact on the phenotype of accelerated aging, which is an interesting hypothesis for further testing.

Cognitive decline during aging is associated with a reduction in the cortical volume, which is closely related to synapse loss [52,53]. The results of the NOR test combined with the Western blotting data for cerebral cortex synaptic markers suggest that Glo1 OEX helped to preserve cognitive function during aging. We found that the levels of some synaptic markers, such as SYP and SNAP25, were elevated in the Glo1 OEX mice, correlating with improved spatial memory performance in both male and female mice. We can speculate that this better performance in the novel object recognition test is, at least in part, due to improvements in synapse connectivity.

Aging impairs mitochondrial dynamics by impairing biogenesis and mitophagy, ultimately disturbing cellular energy homeostasis [54,55]. The preservation in the cerebral cortex of a marker of complex I in males and of a marker of complex II in both males and females may indicate that Glo1 has protective effects on the ETC during aging.

We also looked at proteins associated with protection against the oxytosis/ferroptosis pathway that are downstream of Nrf2 activation, including NQO1, FTH1, and GPx4. We found that the levels of NQO1, FTH1, and GPx4 were significantly upregulated in the brains of the Glo1 OEX SAMP8 mice. These oxytosis/ferroptosis-associated proteins protect cells by distinct mechanisms. Iron accumulation in the brain during aging has been associated with oxidative stress leading to neuronal damage and dendritic spine loss [56], which occurs in specific brain regions, such as the hippocampus and cortex [57]. During aging, increased ferritin levels have been observed in response to elevated iron [58], providing an increase in the iron storage capacity and thus preventing iron toxicity and oxytosis/ferroptosis.

NQO1 reduces quinones by a two-electron process. The hydroquinone formed in the two-electron catalysis avoids the formation of the one-electron radical cycling semiquinone [59]. In a screening of more than 900 natural compounds, quinones showed prominent anti-oxytotic/ferroptotic activity, which was mediated by NQO1 [60]. Moreover, NQO1 induction is widely regarded as participating in antioxidant protection induced by the transcription factor Nrf2 [61].

GPx4 is considered the main enzyme capable of reducing lipid hydroperoxides in membranes, displaying potent anti-oxytotic/ferroptotic action [62]. A number of neurodegenerative diseases are associated with increased cell death by oxytosis/ferroptosis, where the decreased function of GPx4 is frequently observed [62,63]. As a master regulator of membrane lipid peroxidation, increased levels of GPx4 are likely protective to brain cells. Overall, there is a clear induction of anti-oxytotic/ferroptotic proteins (GPx4, FTH1 and NQO1) whose mechanisms of action are distinct, indicating that Glo1 OEX SAMP8 mice present additional protection against a specific cell death mechanism, highlighting once more the benefits of increased levels of Glo1 in the brain.

In certain cases of short bowel syndrome, D-lactate can reach a 3 mM concentration or higher in plasma [64]; however, the main route for D-lactate formation is through glyoxalase metabolism of SDL, the product of Glo1 and the substrate of Glo2 [12,65,66]. In the absence of Glo2, widespread protein lysine lactylation was observed, especially on glycolytic enzymes [66]. One can conclude that increased Glo1 activity is likely to increase its product, SDL, leading to increased DKla in proteins. Glo1 OEX mice presented increased DKlac in nuclear proteins at around 17 kD, most likely representing H3 D-lactylation. This finding opens up the possibility of exploring how MGO metabolism can impact gene expression, a subject that has not been previously studied. Histone deacetylases 1-3 (HDAC1-3) present L- and D-delactylase activity, suggesting the existence of protein site-specific regulatory pathways [67], reinforcing the functional importance of this post-translational modification. Thus, Glo1 OEX against the SAMP8 background represents a unique model to study how DKla participates in gene expression in the context of aging by exploring the age-accelerated phenotype of SAMP8 mice. We acknowledge that additional experiments such as immunoprecipitation of histones before probing for DKla would help to confirm these findings.

## 5. Conclusions

The original idea of overexpressing Glo1 against the SAMP8 background was based on the observation that these mice express significantly lower Glo1 levels in the brain, as compared to control mice as they age. This observation was confirmed here, as we found that multiple tissues present the same low levels of Glo1. The literature showing the negative effects of MGO during aging and in neurodegenerative diseases also highlighted the potential for protective effects of Glo1 overexpression, which is supported by these studies. Moreover, the Glo1-overexpressing SAMP8 mice appear to be normal, lacking deleterious alterations in blood-borne clinical indexes, locomotor activity, limb strength, and clinical histopathology. The frequency of severe visual impairment in males dropped from 20% to 3.2%. Furthermore, Glo1 overexpression promoted an increase in the median survival, especially in males, which was associated with better memory, as shown with the novel object recognition test. This improved memory is likely related to strengthened synapses, as synaptic markers (SNAP25 and SYP) presented elevated levels as compared to WT controls. A marked increase in anti-oxytotic/anti-ferroptotic markers (GPx4, NQO1, and FTH1) was also seen, suggesting that Glo1 overexpression might additionally confer resilience to oxytotic/ferroptotic cell death.

The clear beneficial effects of overexpressing Glo1 in an aging model highlight the need for additional work to investigate the signaling mechanisms involved in the observed responses. A particularly important question is how Glo1 activity interacts with oxytosis/ferroptosis since there are no published studies addressing this relationship.

## Data Availability

The original contributions presented in this study are included in the article/Supplementary Materials. Further inquiries can be directed to the corresponding authors.

## Supplementary Materials

The following supporting information can be downloaded at https://www.mdpi.com/article/10.3390/antiox14080946/s1: Supplementary Methods; Figure S1. CAG-cMyc-hGlo1-IRES2-tdTomato was targeted in the ROSA26 locus by homologous recombination; Figure S2. Immunohistochemistry detecting Glo1 in tissues of WT and Glo1 OEX male mice; Figure S3. Biochemical markers were analyzed in the blood of SAMP8 mice overexpressing Glo1; Figure S4. Locomotor activity of SAMP8 mice overexpressing Glo1; Figure S5. Grip force of SAMP8 mice overexpressing Glo1; Table S1. Summary of histopathological findings in 10-month-old SAMP8 WT and Glo1 OEX mice.

## Abbreviations

The following abbreviations are used in this manuscript:

AGEs	advanced glycation end-products
ATP5A	ATP synthase mitochondrial F1 complex subunit alpha
DKlac	D-lactyllysine modification of proteins
ETC	mitochondrial electron transport chain
FTH1	ferritin heavy chain 1
Glo	glyoxalase
GPx4	glutathione peroxidase 4
H3	histone 3
MGO	methylglyoxal
NDUFB8	NADH:ubiquinone oxidoreductase subunit B8
NOR	novel object recognition
NQO1	NAD(P)H quinone oxidoreductase 1
PSD95	postsynaptic density protein of 95 kD
RAGE	receptor for advanced glycation end-products
SDHB	succinate dehydrogenase B
SDL	S-D-lactoylglutathione
SNAP25	synaptosome-associated protein of 25 kD
SYP	Synaptophysin
UQCRC2	ubiquinol-cytochrome c reductase core protein 2
VDAC	voltage-dependent anion channel
WT	Wild type
