# Supplementary material for: Gender-Dependent Cognitive and Metabolic Benefits Due to Glyoxalase 1 (Glo1) Overexpression in Age-Accelerated SAMP8 Mice
Source: Antioxidants (Basel). 2025 Aug 1;14(8):946. doi: 10.3390/antiox14080946 (PMC12382624; doi:10.3390/antiox14080946)
Supplement: Supplementary file 1 [file antioxidants-14-00946-s001.zip › antioxidants-3755071-supplementary.pdf]

antioxidants

Gender-dependent cognitive and metabolic benefits due to glyoxalase 1 overexpression in age-accelerated SAMP8 mice.

Alcir Luiz Dafre [1,2,*], Taketo Taguchi [1], Yelena Dayn[3], Antonio Currais[1], and Pamela Maher [1, *]

[1] Cellular Neurobiology Laboratory, Salk Institute for Biological Studies, CA, 92037, La Jolla, United States. pmaher@salk.edu
[2] Biochemistry Department, Federal University of Santa Catarina, 88040-900, Florianópolis, SC, Brazil. alcir.dafre@ufsc.br
[3] Transgenic Core Facility, Salk Institute for Biological Studies, CA, 92037, La Jolla, United States
* Correspondence: alcir.dafre@ufsc.br; pmaher@salk.edu

Supplementary Material

Supplementary Methods

For blood biochemical analysis, mice were anesthetized and their blood collected by cardiac puncture and analysis performed by IDEXX BioAnalytics (Comprehensive Chemistry panel, West Sacrament, CA). The endpoints tested included: alanine (ALT) and aspartate (AST) aminotransferase, alkaline phosphatase (ALP), creatin kinase, glucose, cholesterol, bicarbonate, phosphorous, blood-urea nitrogen (BUN), total protein, albumin and globulin.

Locomotor activity was evaluated in the open field using the MED Associates hardware and the Activity Monitor software according to the manufacturer's instructions (MED Associates Inc, St. Albans, VT). Animals were individually placed into clear Plexiglas boxes (40.6 × 40.6 × 38.1 cm) surrounded by multiple bands of photo beams and optical sensors that measure horizontal and vertical activity. Their movement was detected as breaks within the beam matrices and automatically recorded for 30 minutes.

For the histopathology analysis, the animals were perfused, necropsied, and tissue samples were obtained and fixed in formalin. Tissues analyzed included kidney, heart, liver, brain, lungs, spleen, and skeletal muscle. After formalin fixation, the tissues were paraffin-embedded and cut into 5 ◎m sections for standard Hematoxylin and Eosin staining. Images were submitted for evaluation and the findings scored on a scale from 0 to 5 (0=within normal limits, 1= minimal findings or the least change discernible, 2= mild findings, 3=moderate, 4=marked, and 5=severe.

For the immunohistochemistry, the tissues were trimmed, processed, and embedded as formalin-fixed paraffin embedded blocks according to Revela Bioscience's (San Diego, CA) standard protocol. Glo1 antibody (Thermo-Fisher PA5-77987) was used for detection. Anti-Glo1 was detected using Novacast Bond Refine Polymer Detection and visualized with 3'3-diaminobenzidine and hematoxylin used as nuclear counterstain

Fore- and hindlimb grip force was evaluated using an animal grip strength system (San Diego Instruments) with the mice placed over a metal mesh grid connected to a force transducer. After mice gripped the grid with either their fore- or hindlimbs, mice were pulled until the grid was released, and maximal strength recorded, as previously described (doi:10.1016/j.physbeh.2013.11.010.)

Supplementary results

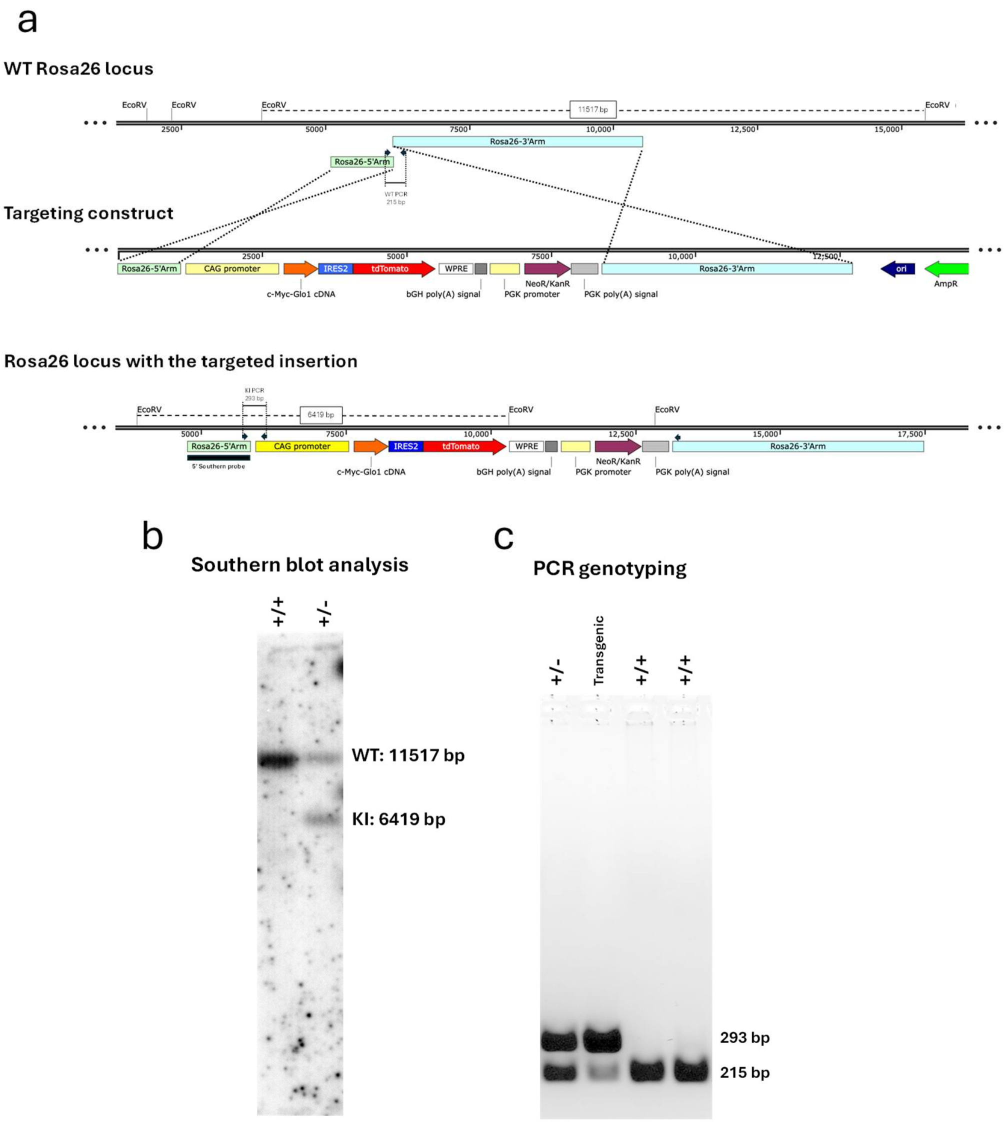

Figure S1. CAG-cMyc-hGlo1-IRES2-tdTomato was targeted into the ROSA26 locus by homologous recombination. (**a**) Diagram of the targeting construct and the expected recombination events. WPRE, the woodchuck hepatitis virus posttranscriptional regulatory element; NeoR/KanR, neomycin/kanamycin resistance positive selection cassette. (**b**) Southern blot analysis, genomic DNAs were digested with EcoRV restriction enzyme and detected by the 5′ arm probe. The size of the WT allele is 11517 bp, the size of the targeted allele is 6419 bp. **(c)** PCR analysis has shown the size of the WT allele is 215 bp and the size of the targeted allele is 293 bp.

a

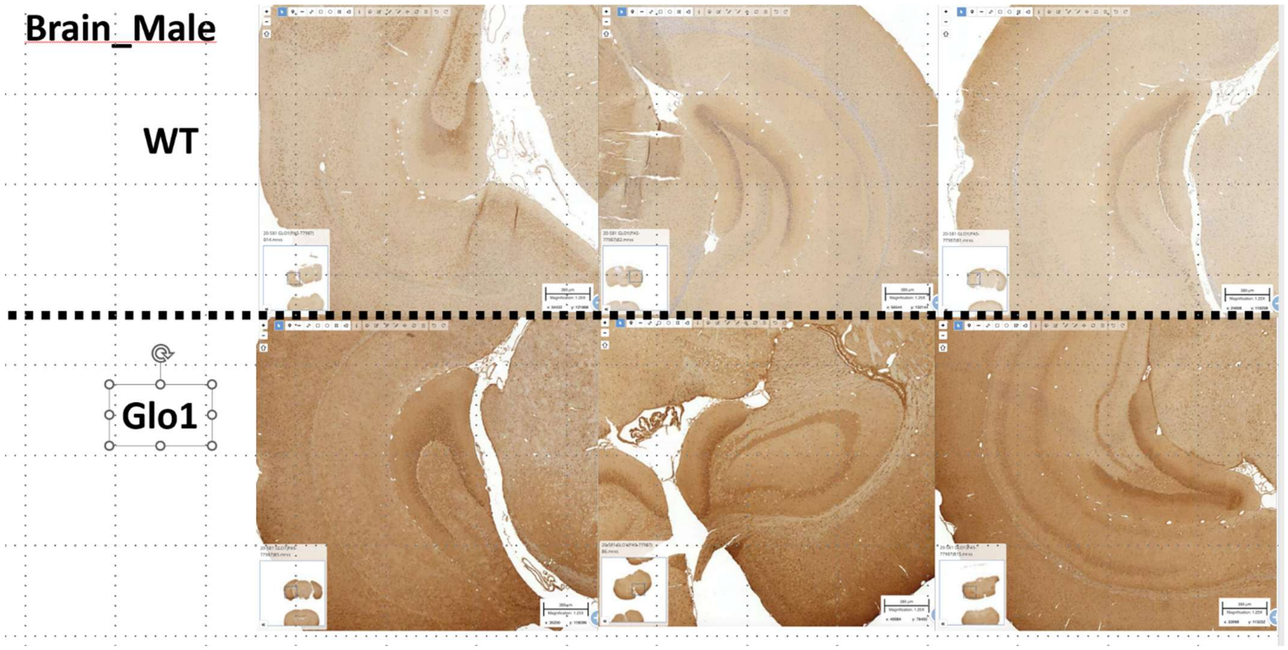

b

Sup. Fig. 3 continues

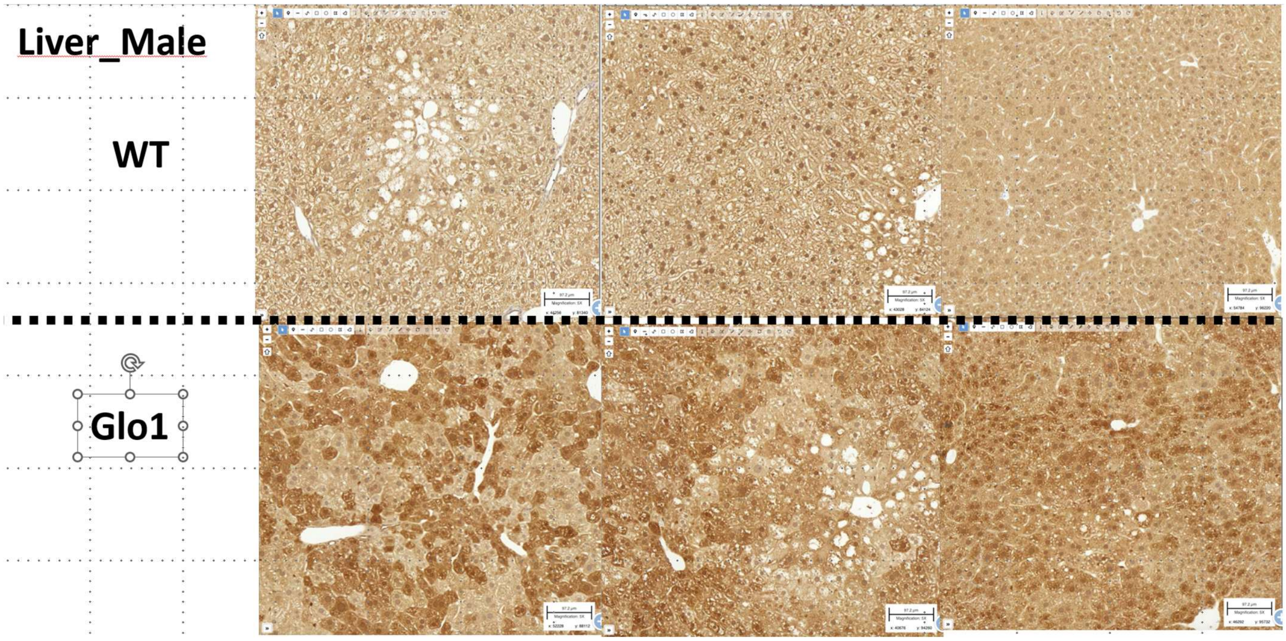

c

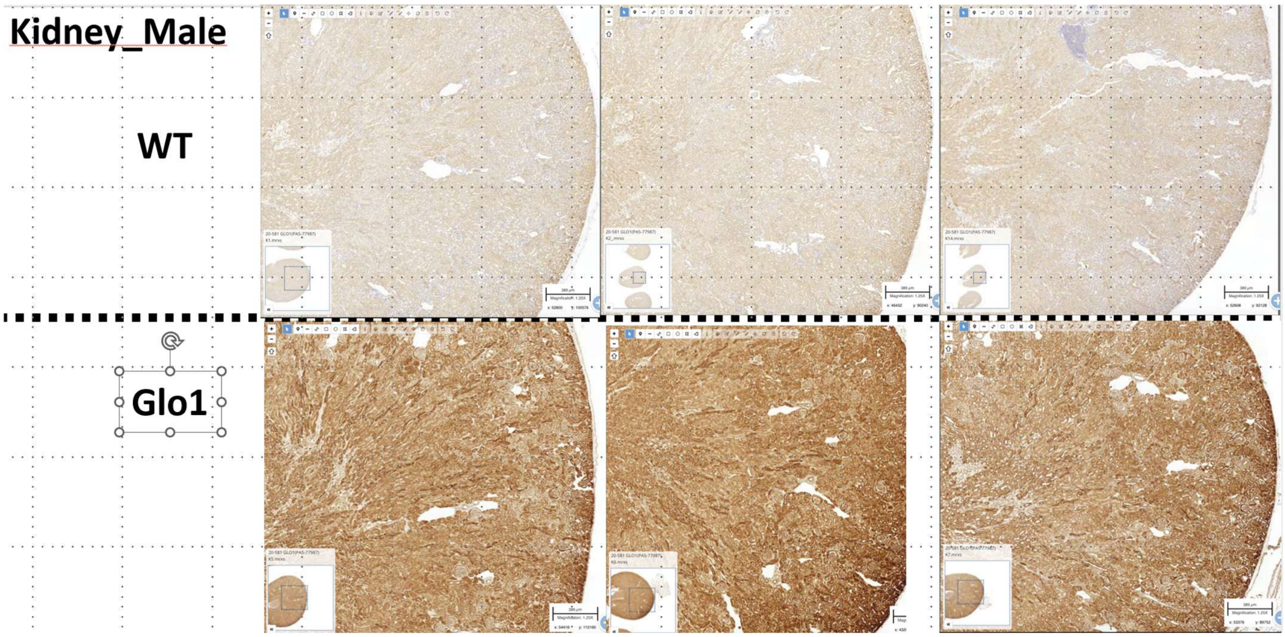

d

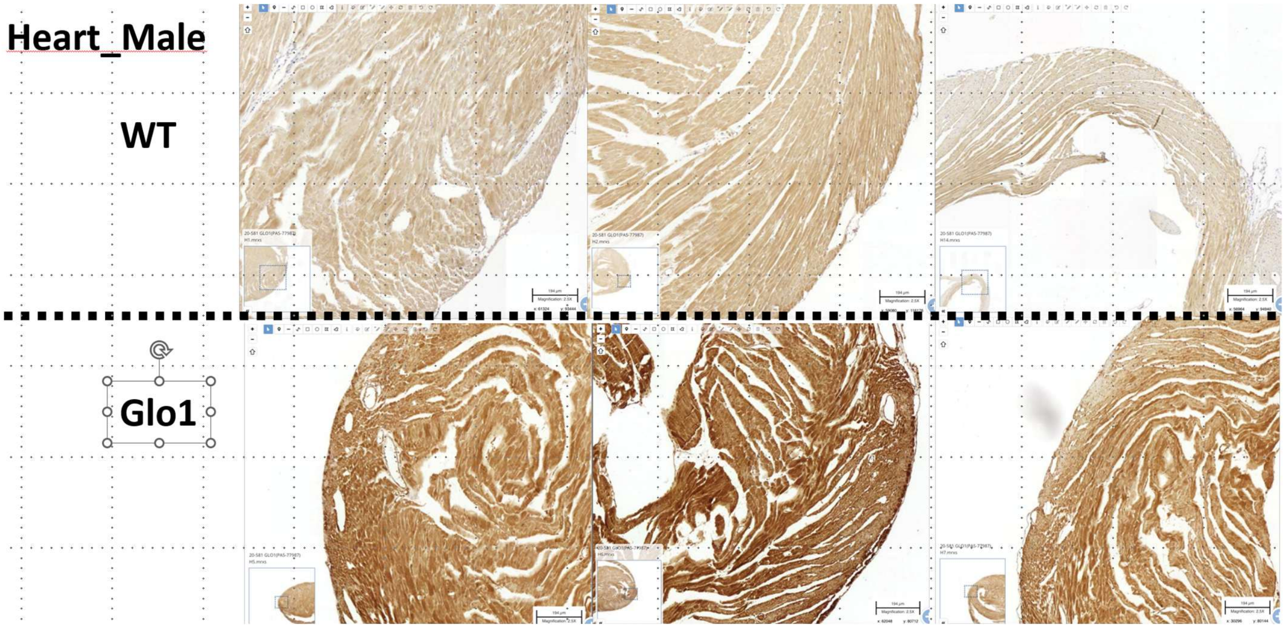

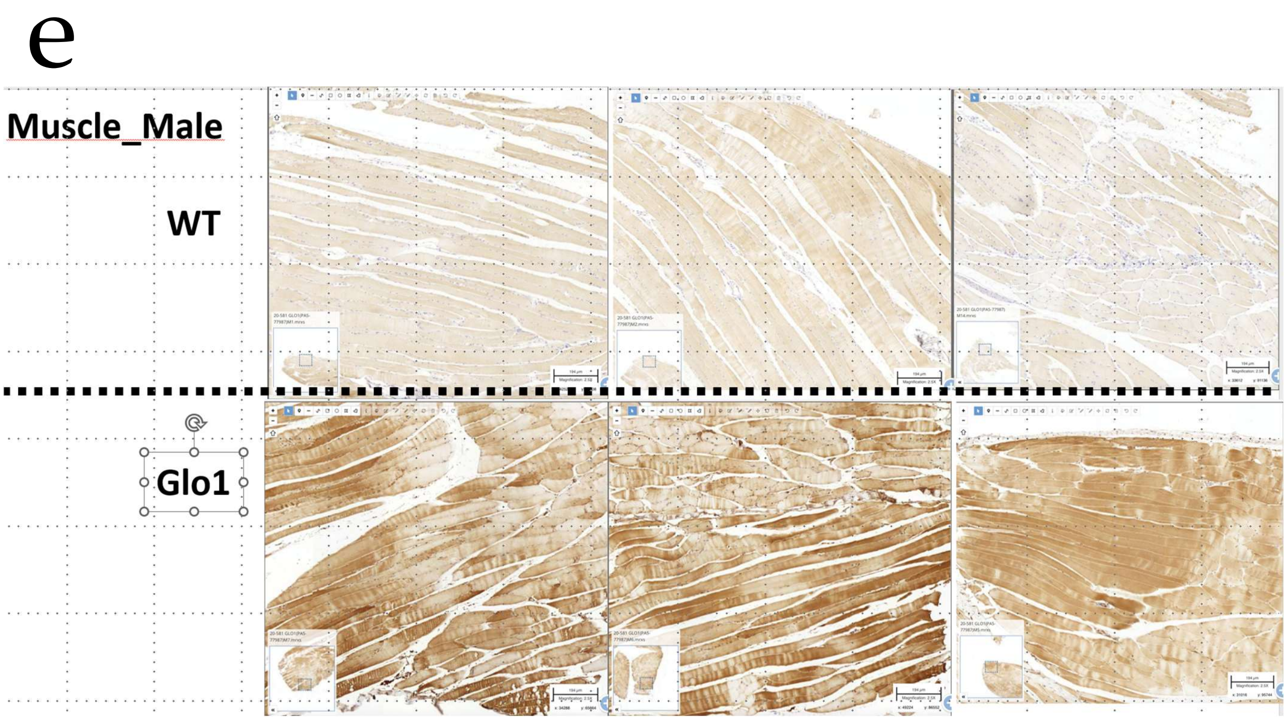

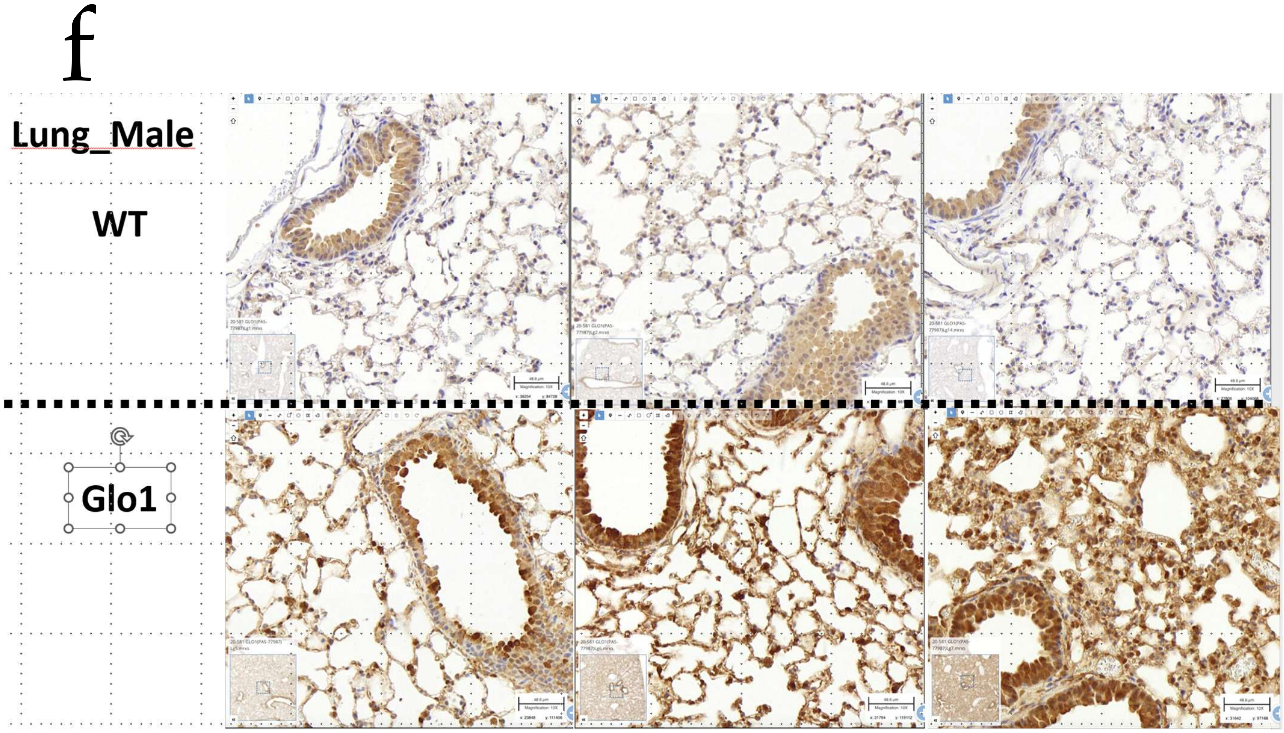

Figure S2. Immunohistochemistry detecting Glo1 in tissues of WT (upper panels) and Glo1 OEX (lower panels) SAMP8 male mice. Three representative images are presented for: (**a**) brain; (**b**) liver; (**c**) kidney; (**d**) heart, (**e**) skeletal muscle, and (**f**) lung.

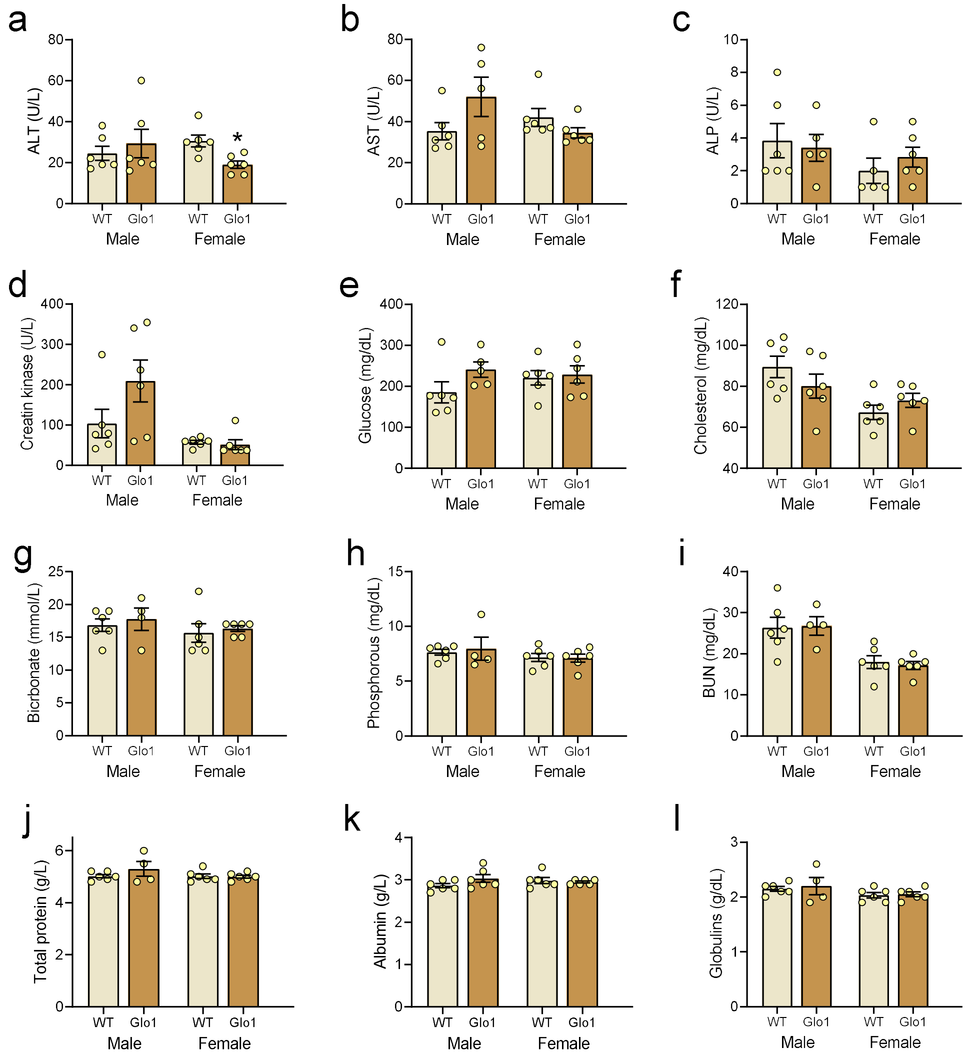

Figure S3. Biochemical markers were analyzed in the blood of WT SAMP8 mice and mice overexpressing Glo1. (**a**) Alanine aminotransferase (ALT), (**b**) aspartate aminotransferase (AST), (**c**) alkaline phosphatase (ALP), (**d**) creatine kinase, (**e**) glucose, (**f**) cholesterol, (**g**) bicarbonate, (**h**) phosphorous, (**i**) blood urea nitrogen (BUN), (**j**) total protein, (**k**) albumin, and (**l**) globulins. Data are presented as mean ± SEM (N=6). Only ALT in female Glo1 OEX mice showed a statistical difference at * $p < 0.05$

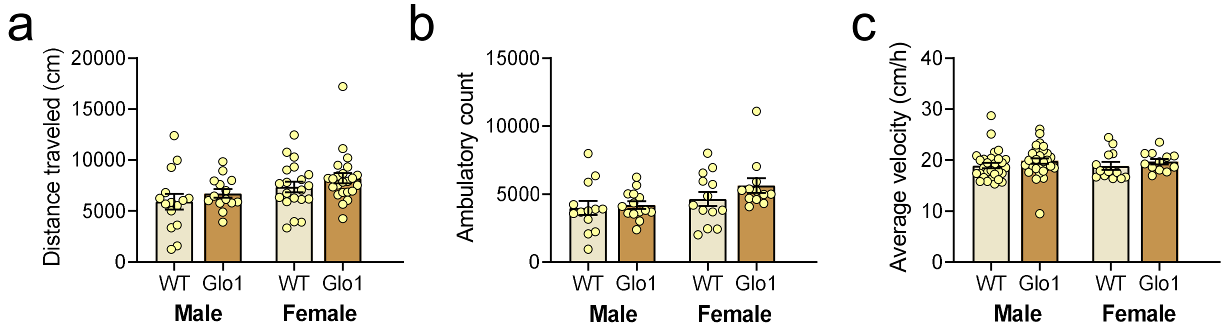

Figure S4. Locomotor activity of WT SAMP8 mice and mice overexpressing Glo1. Male and female mice were evaluated in the open field for locomotor activity at 10-month of age. (**a**) Distance traveled (N=14-15 for male and 20-23 for females); (**b**) Ambulatory crossings on light beam (N=12-14); and (**c**) average velocity (N=29-30 for males and 12-13 for females). Data are presented as mean ± SEM. No statistical differences were found.

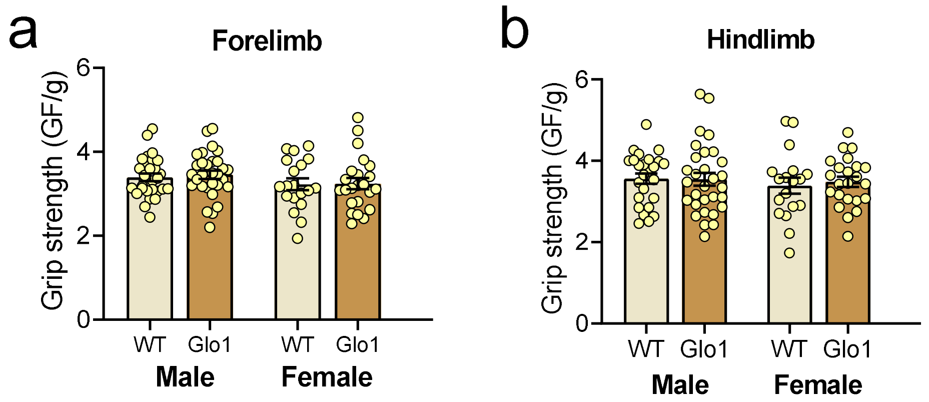

Figure S5. Grip strength of WT SAMP8 mice and mice overexpressing Glo1. Male (N=29-30) and female (N=19-23) mice were tested for grip strength at 10-month of age with the forelimb (**a**) and hindlimb (**b**). Data are presented as mean ± SEM. No statistical differences were found.

Table S1. Summary of histopathological findings in 10 month old SAMP8 WT and Glo1 OEX mice.

**Tissue/Endpoints**	**Male**		**Female**	
	**WT**	**Glo1**	**WT**	**Glo1**
**Brain**				
Occurrences	0/3*	0/3	0/3	0/3
**Kidney**				
Focal perivascular leukocytes	1/3	0/3	2/3	2/3
**Heart**				
Focal chronic inflammation	1/3	0/3	2/3	2/3
**Liver**				
Fatty	3/3	2/3	0/3	1/3
**Lung**				
Lymphoid follicle hyperplasia	2/3	2/3	3/3	1/3
Hemosiderin	1/3	1/3	3/3	1/3
**Muscle**				
Focal leukocytes	0/3	2/3	3/3	1/3
Focal regeneration	0/3	0/3	2/3	2/3
**Spleen**				
Lymphoid hyperplasia	3/3	3/3	3/3	3/3
Hematopoietic hyperplasia	0/2	0/3	1/3	1/3

* Values are the number of animals presenting with the occurrence/total.
